# Ultrasound-based radiomics combined with immune status to predict sentinel lymph node metastasis in primary breast cancer

**DOI:** 10.1038/s41598-023-44156-w

**Published:** 2023-10-07

**Authors:** Miaomiao Zhao, Yan Zheng, Jian Chu, Zhenhua Liu, Fenglin Dong

**Affiliations:** 1 Department of Ultrasound, The Yancheng Clinical College of Xuzhou Medical University, The First People’s Hospital of Yancheng, 66 Renmin Road, Yancheng, 224005 China; 2 Department of Ultrasound, The First Affiliated Hospital of Soochow University, 899 Pinghai Road, Suzhou, 215000 China; 3 Department of General Surgery, The Yancheng Clinical College of Xuzhou Medical University, The First People’s Hospital of Yancheng, 66 Renmin Road, Yancheng, 224005 China; 4 Department of Radiotherapy, The Yancheng Clinical College of Xuzhou Medical University, The First People’s Hospital of Yancheng, 66 Renmin Road, Yancheng, 224005 China

**Keywords:** Breast cancer, Cancer imaging, Tumour biomarkers

## Abstract

In the past few years, the axillary lymph node dissection technique has been steadily replaced by sentinel lymph node biopsy for treating and diagnosing breast cancer, thereby minimizing the complications and sequelae of the patients. Nevertheless, sentinel lymph node biopsy still presents limitations, such as high operation requirements, prolonged surgical duration, and adverse reactions to tracer agents. This study developed a novel non-invasive method to predict sentinel lymph node metastasis in breast cancer by analyzing the ultrasound imaging characteristics of the primary tumor, combined with the analysis of peripheral blood T-cell subsets that reflect the immune status of the body. The radiomic features analyzed in this study were extracted from preoperative ultrasound images of 199 solitary breast cancer patients, who were undergoing surgery and were pathologically diagnosed at the Yancheng First People's Hospital. All cases were randomly categorized in a 4:1 ratio to the training (n = 159) and validation (n = 40) cohorts. The extracted radiomics features were subjected to dimensional reduction with the help of the least absolute shrinkage and selection operator technique, resulting in the inclusion of 19 radiomics features. Four classifiers, including naïve Bayesian, logistic regression, classification decision tree, and support vector machine, were utilized to model the radiomics features, conventional ultrasound features, and peripheral blood T cell subsets in the training dataset, and validated using the validation dataset. The best-performing model was chosen for constructing the combined model. The radiomics model constructed using the logistic regression showed the best performance, with the training and validation cohorts showing areas under the curve (AUCs) of 0.77 and 0.68, respectively. The conventional ultrasound and peripheral blood T cell models constructed using the classification decision tree showed the best performance, wherein the training cohort presented AUCs of 0.71 and 0.81, respectively, while the validation cohort presented AUCs of 0.68 and 0.69, respectively. The combined model constructed by logistic regression showed AUCs of 0.91 and 0.79 in the training and validation datasets, respectively. The resulting combined model can be considered a simple, non-invasive method with strong reproducibility and clinical significance. Thus, it can be utilized to predict sentinel lymph node metastasis in breast cancer. Furthermore, the combined model can be effectively used to guide clinical decisions related to the selection of surgical procedures in breast surgery.

## Introduction

Breast cancer is the most common malignancy in women. The International Agency for Research on Cancer (IARC) of the World Health Organization (WHO) published the recent global cancer burden data for 2020, in June 2021. The data indicated that the incidence of breast cancer in the world increased to 2.26 million cases, exceeding the 2.2 million lung cancer cases to become the highest-incidence cancer type across the world^1^.

The most common site of breast cancer metastasis is reported to be the axillary lymph nodes, and numerous studies have shown that the patients with positive axillary lymph node involvement presented a 5-year disease-free survival rate that was 20% lower than that displayed by the patients with negative involvement^2^. Axillary lymph node dissection (ALND) is a very effective way to treat axillary lymph node metastasis in breast cancer. However, due to significant complications and sequelae, the sentinel lymph node biopsy (SLNB) technique, which is safer and less invasive, has eventually replaced ALND as the preferred approach for evaluating axillary lymph node metastasis in breast cancer. The SLNB results in the axilla can predict whether the axillary lymph nodes are metastatic, and show an accuracy of > 90%. However, SLNB also has some drawbacks. Firstly, the process involving the localization of the sentinel lymph node with a "tracer agent" is accompanied by side effects such as infection, allergies, skin staining, and radiation damage. Secondly, the improvement of the accuracy in the process requires excising three or more sentinel lymph nodes^3–6^. This process is also associated with more complex surgical procedures, longer surgical times, and higher surgical trauma and risks.

According to the growth characteristics of malignant tumors, the sentinel lymph node metastasis shows a moderate correlation with a few features that reflect the degree of malignancy of the tumor, such as location^7–9^, size^9–11^, margin^11,12^, and echogenicity^13^. The characteristics of the primary tumor in breast cancer can serve as potential markers for predicting sentinel lymph node metastasis. In a 2012 Dutch study, Lambin et al. first proposed the concept of radiomics^14^. Radiomics makes use of machine learning techniques to quantitatively analyze medical images and associate them with different clinical and genetic features of patients. It has significantly improved precision medicine and helped in differentiating between malignant and benign tumors in various types of cancers. Currently, radiomics has made significant advances in breast cancer screening and the study of molecular subtyping and axillary lymph node metastasis^15–20^.

As malignant tumors develop, they make use of an immune editing process to suppress the recognition and killing of tumor cells by the immune system. Recent research has confirmed that cancer cells that metastasize to lymph nodes can further stimulate the differentiation and proliferation of regulatory T cells (Tregs), thus creating an immune-suppressive environment in the body that is more conducive to the metastasis and colonization of cancer cells^21^. It was noted that T cells, which were crucial immune cells, play a crucial role in the immune responses of the body against tumors. The subpopulation structure of peripheral blood T cells can reflect the anti-tumor immune level of the organism. Thus, it can be observed that there is a correlation between the structural phenotype of peripheral blood T cell subsets and sentinel lymph node metastasis in breast cancer.

Herein, a combined model by analyzing the radiomic features of primary tumors in breast cancer to assess the peripheral blood T cell subsets, which reflect the overall immune status. This study aimed to explore a new predictive method for predicting sentinel lymph node metastasis in breast cancer patients, to optimize the clinical applications of the SLNB procedures.

## Materials and methods

### Data acquisition

The study data were derived from a retrospective observational study that followed the STROBE guidelines.

This study included 199 patients with solitary breast cancer who were diagnosed through postoperative pathological examination, had complete preoperative peripheral blood T cell subset analysis data, and underwent concurrent SLNB at the Yancheng First People's Hospital between October 1, 2020, and December 31, 2022. The patients meeting the following inclusion criteria were examined in this study: (1) Patients with solitary breast cancer without distant metastasis; (2) Patients who underwent SLNB and had a clear pathological diagnosis; (3) Patients with complete pre-treatment ultrasound imaging data of the primary lesion of breast cancer, with clearly visible lesions; (4) Patients with complete clinical data and T cell subset analysis results.

**Figure 1:**
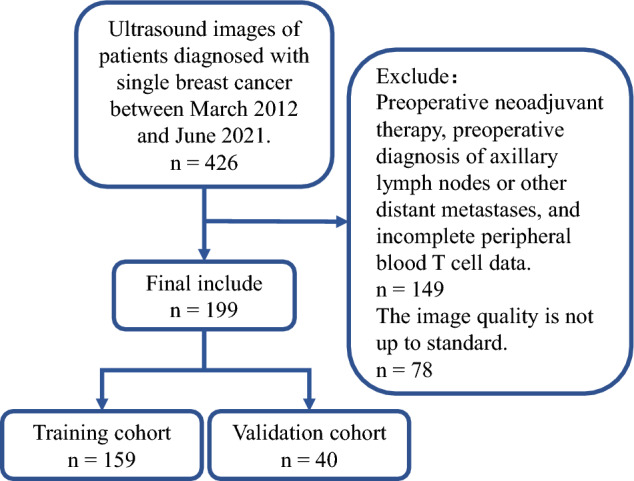
Flow diagram of the recruitment pathway.

All 199 cases included in this study were divided randomly into the training (n = 159) and validation (n = 40) cohorts in a 4:1 ratio. The clinical features of all the included cases are presented in Table 1. Figure 1 presents the patient selection process.

**Table 1:** Clinical and Ultrasonic characteristics of primary breast cancer. *P* values were calculated by Student’s *t* test or Fisher’s exact test between Training cohort and Test cohort, where appropriate. SLNM means sentinel lymph node metastasis. Values are mean ± SD or no. (%). IDC means invasive ductal carcinoma.

Variable	Training cohort (n = 159)	Validation cohort (n = 40)	*P* value
Age (Year)	56.7 ± 14.2	55.5 ± 10.8	0.61
Major axis (mm)	23.7 ± 12.6	23.8 ± 12.5	0.98
Minor axis (mm)	15.5 ± 7.3	15.2 ± 7.6	0.81
Area (mm^2^)	334.2 ± 353.9	325.7 ± 317.7	0.76
SLNM			1.0
Positive	53 (33%)	13 (33%)	
Negative	106 (67%)	27 (67%)	
Pathology			0.61
IDC	103 (65%)	35 (62%)	
Others	56 (35%)	15 (38%)	
ER			0.1
Positive	100 (63%)	19 (48%)	
Negative	59 (37%)	21 (52%)	
PR			0.15
Positive	81 (51%)	15 (38%)	
Negative	78 (49%)	25 (62%)	
Her-2			0.86
Positive	61 (38%)	16 (40%)	
Negative	98 (62%)	24 (60%)	
Parallel to skin ratio	1.6 ± 0.6	1.7 ± 0.9	0.55
Quadrant			0.66
Inner	129 (81%)	31 (78%)	
Outer	30 (19%)	9 (22%)	
Shape			0.66
Regular	6 (4%)	2 (5%)	
Irregular	153 (96%)	38 (95%)	
Margin			0.69
Smooth	10 (6%)	1 (3%)	
Burr/Lobular/angulate	149 (94%)	39 (97%)	
Calcification			1.0
Negative	74 (47%)	19 (48%)	
Positive	85 (53%)	21 (52%)	
Echo			1.0
Mixed	19 (12%)	4 (10%)	
Low	140 (88%)	36 (90%)	
CD3^+^	1016.2 ± 266.2	974.3 ± 292.2	0.38
CD8^+^	369.2 ± 119.4	347.7 ± 105.6	0.3
CD4^+^	525.8 ± 139.6	512.4 ± 119.8	0.58
CD4/CD8	1.5 ± 0.5	1.6 ± 0.4	0.74

### Ultrasound image acquisition

The ultrasound diagnostic instruments used in this study included PHILIPS EPIQ 5, GE LOGIQ E9, and TOSHIBA Aplio 500. The probe models were L12-3 (PHILIPS EPIQ 5), ML6-15-D (GE LOGIQ E9), and 14L5 (TOSHIBA Aplio 500).

For analysis, the patients were placed in a supine position with both hands raised, fully exposing both breasts and the axillary region, 1–2 weeks before surgery. Longitudinal, transverse, and radial scans centered around the nipples were conducted to examine both breasts, where the breast lesions were scanned at multiple angles. Finally, a scan of both axillary regions was performed. The acquired images were collected and preserved in the DICOM format.

### Analysis of peripheral blood T cell subsets

The patients included in the study had their fasting blood samples collected one week before surgery. The samples were processed using a four-color lymphocyte subset reagent kit, which was compatible with the EPICS XL flow cytometer (Beckman Coulter, CA, USA). The peripheral blood T cell subsets were detected using the flow cytometer, while the data were analyzed using the accompanying software.

### Image processing and segmentation

**Figure 2:**
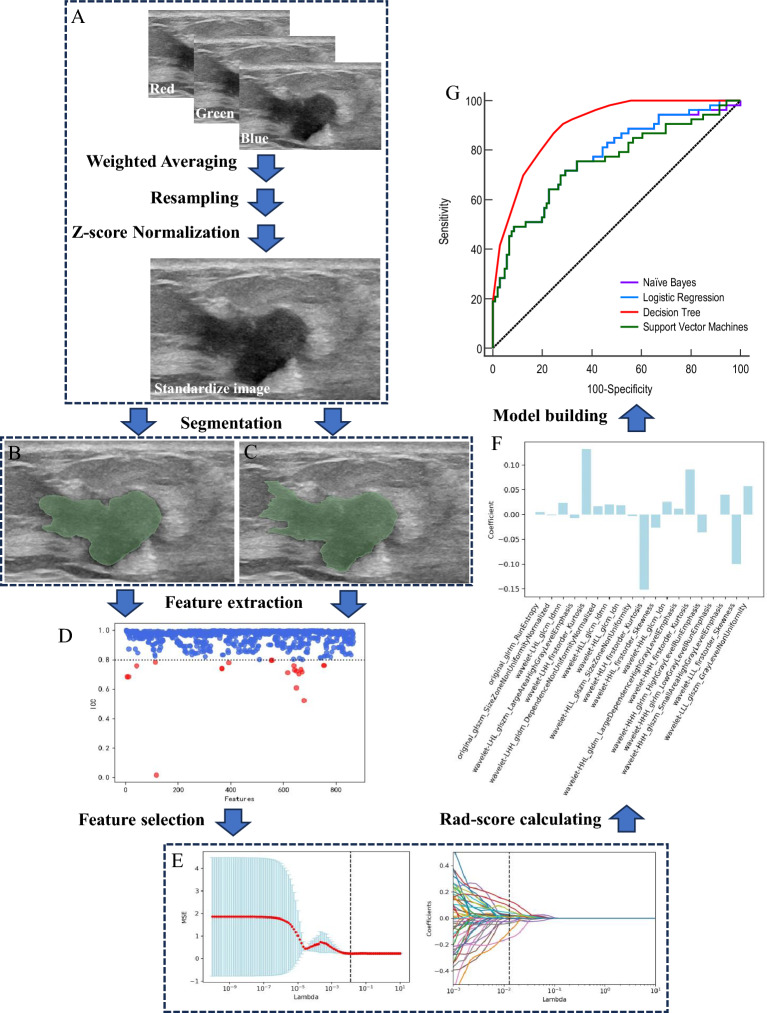
Workflow of radiomics model building and analysis. To standardize the images, all of the ultragraphs were preprocessed using Weighted Averaging, Resampling, and Z-score Normalization (**A**). Then standardized images were segmented by two ultrasound physicians independently (**B**, **C**). 864 radiomics features were extracted from each segmentation, and the consistency of the two sets of radiomics features was initially assessed using ICC (**D**). The least absolute shrinkage and selection operator (LASSO) was used to select the features (**E**). The radiomic scores for each case were calculated using these 19 radiomic features (**F**). The radiomics model was constructed using logistic regression, naïve Bayes, support vector machine, and classification decision tree methods in the training cohort (**G**).

Each region of interest (ROI) was independently delineated by two ultrasound physicians using 3DSlicer (Version 4.11.0), who had no prior knowledge of whether the patient had sentinel lymph node metastasis. Both ultrasound physicians had 5 to 10 years of ultrasound diagnostic experience. As shown in Fig. 2, all included images were reconstructed into grayscale images using a weighted average method, and each voxel was resized to 1 mm × 1 mm × 1 mm using linear interpolation. Finally, the images were subjected to Z-score normalization.

### Feature extraction and selection

The open-source PyRadiomics toolkit (ver. 3.0) in Python (ver. 3.7) was used for extracting the radiomic features from 2 sets of ROIs. The extracted features primarily included first-order statistical features, tumor morphological features, texture features, and wavelet features. Furthermore, the interclass/intraclass correlation coefficient (ICC) was utilized in the training cohort to assess the consistency between the two sets of radiomic features. Features with an ICC ≥ 0.8 were subjected to the Mann–Whitney U test for initial screening. After normalizing the radiomic data using the Z-score method, a feature dimension reduction was carried out using the least absolute shrinkage and selection operator with a cross-validation (LASSO-CV) algorithm, yielding the calculation of radiomic scores (Rad-score).

### Model construction and validation

The following parameters were used in this study; long diameter, short diameter, margin, echogenicity, calcification, aspect ratio from ultrasound images of primary tumors in breast cancer, peripheral blood CD3^+^, CD4^+^, CD8^+^ T cell counts, CD4/CD8 ratio, and radiomic scores. In the training cohort, four methods, namely support vector machine, logistic regression, naïve Bayes, and classification decision tree, were employed to construct conventional ultrasound prediction models. Each model was assessed with the help of the receiver operating characteristic (ROC) curves in the training and validation cohorts to compare their predictive performances. The model exhibiting the best performance was selected, and a combined model was established using logistic regression. Then, the performance of this combined model was validated with the validation cohort.

### Ethics statement

The study protocol was performed in accordance with the guidelines outlined in the Declaration of Helsinki. The Ethics Committee of The First people’s Hospital of Yancheng approved the study (2023-K-102), and all participants signed informed consent statements.

## Results

### Clinical features

No statistically significant differences were observed between the training and validation cohorts in terms of different factors like age, histological subtype, molecular subtype, primary tumor size, conventional ultrasound features, and peripheral blood T cell subset structure (Table 1).

### Selection of radiomic features

Herein, both physicians used the PyRadiomics toolkit (ver. 3.0) to extract the radiomic features from the delineated ROIs. These features primarily included first-order statistical features, tumor morphological features, texture features, and wavelet features, yielding a total of 864 radiomic features. The two sets of radiomic features in the training cohort were first subjected to consistency analysis using the ICC. Out of the 841 features with an ICC ≥ 0.8, 81 features with statistically significant differences were selected through the Mann–Whitney U test. Furthermore, the LASSO-CV was used for dimension reduction of the aforementioned 81 features based on fivefold cross-validation, resulting in a final set containing 19 radiomic features (Fig. 2). The radiomic scores for each case were calculated using these 19 radiomic features and presented in Table 2.

**Table 2:** The final features and coefficients were selected for radiomics-score calculating.

Coefficients	Features
+ 0.013499660178093648	original_glrlm_RunEntropy
− 0.03666887276677416	original_glszm_SizeZoneNonUniformityNormalized
+ 9.536611440593765	wavelet-LHL_glcm_Idmn
− 6.565758503646565e-11	wavelet-LHL_glszm_LargeAreaHighGrayLevelEmphasis
+ 0.001029211440995142	wavelet-LHH_firstorder_Kurtosis
+ 0.6050819244953135	wavelet-LHH_gldm_DependenceNonUniformityNormalized
+ 9.01127105142491	wavelet-HLL_glcm_Idmn
+ 2.274891015307939	wavelet-HLL_glcm_Idn
− 3.939196203052282e-5	wavelet-HLL_glszm_SizeZoneNonUniformity
− 0.0009733962921809391	wavelet-HLH_firstorder_Kurtosis
− 0.009440558947116654	wavelet-HHL_firstorder_Skewness
+ 2.286789125371559	wavelet-HHL_glcm_Idn
+ 2.4507545186318116e-5	wavelet-HHL_gldm_LargeDependenceHighGrayLevelEmphasis
+ 0.00035478213502434803	wavelet-HHH_firstorder_Kurtosis
− 13.901905731678827	wavelet-HHH_glrlm_HighGrayLevelRunEmphasis
+ 5.167060464573376e-10	wavelet-HHH_glrlm_LowGrayLevelRunEmphasis
+ 0.08981598802575962	wavelet-HHH_glszm_SmallAreaHighGrayLevelEmphasis
− 0.0552117644951858	wavelet-LLL_firstorder_Skewness
0.0013798056448859272	wavelet-LLL_glszm_GrayLevelNonUniformity
12.157508	Constant

### Establishment and validation of the univariate model

**Figure 3:**
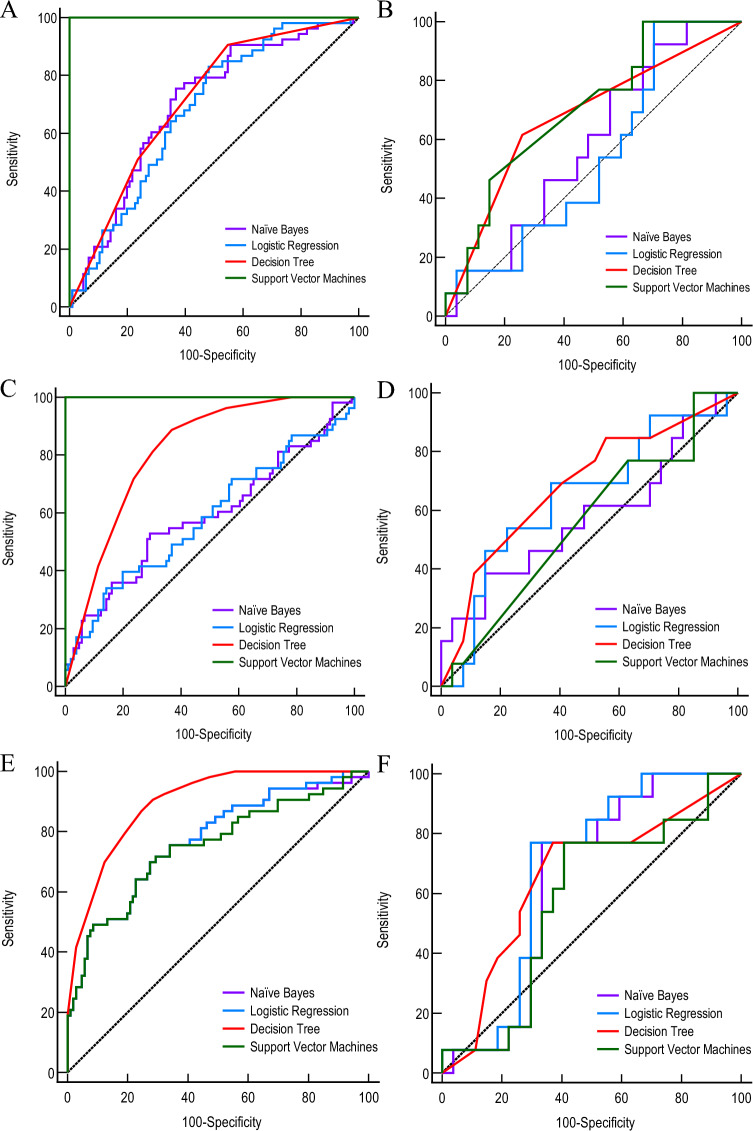
ROC curves of the different conventional ultrasound models in training (**A**) and validation cohorts (**B**), the different peripheral blood T cells models in training (**C**) and validation cohorts (**D**), and the different radiomics models in training (**E**) and validation cohorts (**F**).

The univariate model was constructed using logistic regression, naïve Bayes, support vector machine, and classification decision tree methods in the training cohort. These models were then validated using the validation cohort to identify the best-performing model. The classification decision tree model of conventional ultrasound showed an AUC of 0.71 (95% CI 0.64–0.78) in the training cohort and 0.68 (95% CI 0.51–0.82) in the validation cohort to predict the sentinel lymph node metastases in breast cancer. The classification decision tree model of peripheral blood T cells presented an AUC of 0.81 (95% CI 0.74–0.87) in the training cohort and 0.69 (95% CI 0.52–0.82) in the validation cohort to predict the sentinel lymph node metastases in breast cancer. The logistic regression model of radiomics showed an AUC of 0.77 (95% CI 0.70–0.83) in the training cohort and 0.68 (95% CI 0.52–0.82) in the validation cohort (Fig. 3). Table 3 displays the detailed model performance observed in this study.

**Table 3:** Discriminative performance of different models in training and validation cohorts.

Model	Cohort	Method	AUCs	95% CI	Sensitivity (%)	Specificity (%)
Conventional Ultrasound						
	Training					
		Naïve bayes	0.7	0.62–0.77	75.5	63.2
		Logistic regression	0.68	0.6–0.75	83	51.9
		Decision tree	0.71	0.64–0.78	90.6	45.3
		Support vector machines	1.0	0.98–1.0	100	100
	Validation					
		Naïve bayes	0.58	0.42–0.74	92.3	29.6
		Logistic regression	0.54	0.37–0.7	100	29.6
		Decision tree	0.68	0.51–0.82	61.5	74.1
		Support vector machines	0.7	0.54–0.84	100	33.3
T cell						
	Training					
		Naïve bayes	0.59	0.51–0.67	52.8	70.8
		Logistic regression	0.58	0.5–0.66	39.6	80.2
		Decision tree	0.81	0.74–0.87	88.7	63.2
		Support vector machines	1.0	0.98–1.0	100	100
	Validation					
		Naïve bayes	0.58	0.41–0.73	38.5	85.2
		Logistic regression	0.64	0.48–0.79	69.2	63.0
		Decision tree	0.69	0.52–0.82	84.6	44.4
		Support vector machines	0.56	0.39–0.71	100	14.8
Radiomics						
	Training					
		Naïve bayes	0.77	0.70–0.83	71.7	70.8
		Logistic regression	0.77	0.70–0.83	71.7	70.8
		Decision tree	0.90	0.84–0.94	90.6	71.7
		Support vector machines	0.75	0.68–0.82	71.7	70.8
	Validation					
		Naïve bayes	0.64	0.48–0.79	77.0	66.7
		Logistic regression	0.68	0.52–0.82	76.9	70.4
		Decision tree	0.66	0.49–0.80	76.9	63.0
		Support vector machines	0.58	0.41–0.73	76.9	59.3
Combined Model						
	Training					
		Naïve bayes	0.91	0.86–0.95	88.7	78.3
		Logistic regression	0.91	0.85–0.95	73.6	90.6
		Decision tree	0.94	0.89–0.97	88.7	81.1
		Support vector machines	0.91	0.85–0.95	94.3	74.5
	Validation					
		Naïve bayes	0.72	0.55–0.75	92.3	51.9
		Logistic regression	0.79	0.64–0.90	84.6	74.1
		Decision tree	0.75	0.58–0.87	61.5	85.2
		Support vector machines	0.78	0.62–0.89	92.3	55.6

### Establishment and validation of the combined model

The training cohort was used for constructing the combined model using logistic regression. The model that exhibited the best performance was selected after validation using a different validation cohort. The combined model presented an AUC of 0.91 (95% CI 0.85–0.95), with a sensitivity of 73.6% and specificity of 90.6% in the training cohort. Furthermore, in the validation cohort, the AUC was recorded to be 0.79 (95% CI 0.64–0.90), with an 84.6% sensitivity and 74.1% specificity (Table 3).

**Figure 4:**
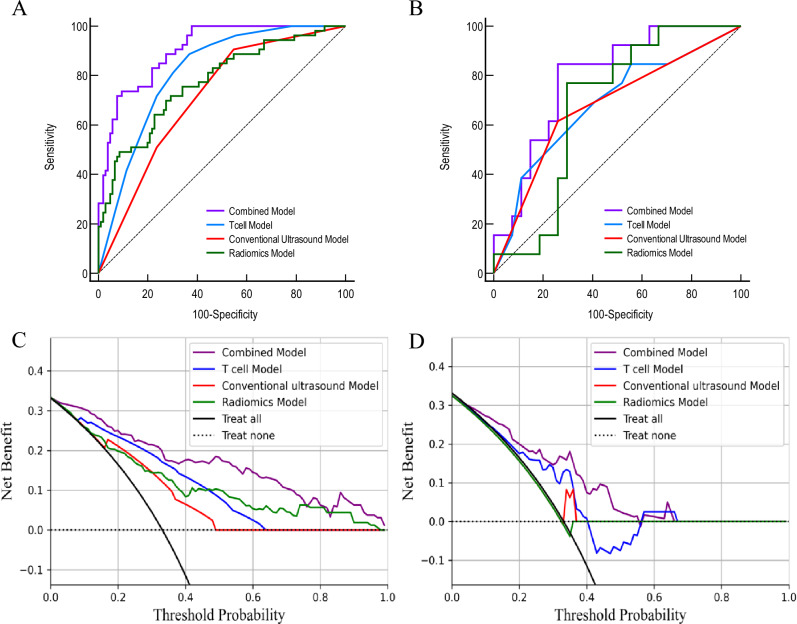
ROC curves of the combined and univariate models in training (**A**) and validation cohorts (**B**). Decision curve analysis of different models in training (**C**) and internal validation cohorts (**D**).

Based on the data derived from the clinical decision curve, it was observed that the combined model had greater clinical application value in predicting the sentinel lymph node metastasis in breast cancer compared to other univariate models (Fig. 4).

## Discussion

There has been a gradual increase in the incidence of breast cancer in women, which establishes it as the most common type of cancer worldwide. Breast cancer accounts for approximately 30% of all cancers in women globally, and the ratio of mortality to incidence is approximately 15%^2^. Patients with breast cancer with axillary lymph node metastasis show a significantly unfavorable prognosis. It was observed that the 5-year disease-free survival rate in patients with positive axillary lymph node metastasis was 20% lower than the value displayed by patients with negative lymph node metastasis^2^. ALND is regarded as the most effective approach for treating axillary lymph node metastasis in breast cancer. However, ALND not only increases surgical trauma for patients but also presents significant complications and sequelae, such as lymphatic leakage, wound infection, edema of the affected upper limb, scar deformities in the neck and axillary regions, and sensory abnormalities. These complications and sequelae have a substantial impact on the following treatment and quality of life of the patients. In recent years, SLNB has gradually replaced ALND as the standard technique used to assess axillary lymph node metastasis in breast cancer^22^. Despite its advantages, SLNB also presents notable limitations. Primarily, the administration of a “tracer agent” molecule to identify the sentinel lymph node is accompanied by adverse effects, including, but not limited to infection, allergic reactions, skin staining, and radiation-induced harm. Moreover, to enhance the precision, it is often necessary to extract 3 or more sentinel lymph nodes^2–4^, which consequently entails intricate surgical procedures, extended operative duration, and increased surgical trauma and risks. In summary, a convenient and non-invasive auxiliary method is urgently needed in clinical practice to provide a basis for surgical decision-making.

Conventional imaging examinations can identify significantly enlarged axillary lymph nodes and evaluate their potential for cancer metastasis based on their morphology, margins, structure, and blood supply. However, the application of “tracer agent” and drainage localization is a necessary step that has to be implemented in patients with clinically negative lymph nodes, to identify the sentinel lymph nodes. If tracer agent imaging is not feasible, the characteristics of the primary breast tumor can be used as a potential marker to predict sentinel lymph node metastasis. Herein, a combined predictive model was constructed by integrating conventional ultrasound imaging and peripheral blood T cell subset analysis to predict the presence of sentinel lymph node metastasis in breast cancer patients. The proposed method offers a non-invasive and localization-free approach, devoid of any long-term consequences to predict the presence of sentinel lymph node metastasis. To construct the model, this study retrospectively collected the data from 426 pathologically-confirmed solitary breast cancer patients, who underwent surgery at the Yancheng First People's Hospital from October 1, 2020, to December 31, 2022. Ultimately, 199 cases were included in the study, with a sentinel lymph node positivity rate of 33%.

In clinical practice, ultrasound diagnosticians distinguish the malignancy degree of primary breast tumors by analyzing the size, morphology, margins, internal echogenicity, and echogenicity of surrounding tissues. In this study, a classification decision tree model demonstrated good predictive performance in conventional ultrasound examinations, with AUCs of 0.71 and 0.68 for the training and validation cohorts, respectively. Fanizzi et al.^23^ constructed a predictive model using clinical information to assess the area under the curve (AUC) for predicting sentinel lymph node metastasis in breast cancer, which yielded an AUC of 0.647. In contrast, the model developed by Bove et al.^24^ achieved an AUC of 0.739. We believe that the study by Fanizzi et al. is based on a larger sample size and has reliable external validation, making their results more reliable. On the other hand, both the study by Bove et al. and our model are based on small sample data and lack reliable external validation. Therefore, the observed favorable performance in our case might be attributed to chance. Indeed, an essential point to highlight is that the two models mentioned above incorporate pathological information, while our model solely relies on traditional ultrasound features of the primary breast tumor. This key distinction underscores the true non-operative nature of our predictive model. These findings in this study implied that the conventional ultrasound model could be utilized to predict the presence of sentinel lymph node metastasis in breast cancer.

Preclinical research has indicated that there is mutual circulation and complementation between peripheral blood T cells and T cells infiltrating tumors^25^. Cancer with lymph node metastasis may present a more obvious immunosuppressive state^26^, which can be reflected in the subset structure of peripheral blood T cells. Clinical studies have also revealed that the subset structure of peripheral blood lymphocytes is related to the prognosis of patients with breast cancer^27^. In this study, the peripheral blood T cell model that was constructed using a classification decision tree exhibited good predictive performance, with an AUC of 0.81 in the training cohort and 0.69 in the validation cohort. These data indicate that the peripheral blood T cell model can accurately anticipate the sentinel lymph node metastasis in breast cancer.

In a 2012 Dutch study, Lambin et al. first proposed the concept of radiomics^14^. Radiomics is used to extract and quantitatively analyze various subtle texture features related to the target in medical imaging data through high-throughput computing to construct prediction models. The ROI serves as the specific object in radiomics research. We referred to similar studies, and based on Bove et al.'s finding, the radiomics model constructed using the original region of interest (ROI) achieved the best performance compared to the intra-tumoral ROI, peritumoral ROI, and combined ROI methods. Therefore, we also employed manual segmentation of the original ROI in our study. Based on the delineation style of the ROI, physician A prefers smoother boundaries (Fig. 2B), which could lead to the inclusion of partial tumor surrounding tissue within the ROI. On the other hand, physician B was prone to clearer and more detailed boundaries (Fig. 2C), which could result in the exclusion of certain tumor tissue. Despite the different styles of the 2 ROI sets, the extracted radiomic features still exhibited an ICC ≥ 0.8 in > 95% of cases(Fig. 2D). This indicates a high consistency in the texture characteristics exhibited by primary breast tumors. The method demonstrates good repeatability and can be applied in clinical practice. The features with good consistency were further screened using the Mann–Whitney U test and LASSO_CV based on fivefold cross-validation (Fig. 2E). Eventually, 19 radiomic features were selected. These features were then used to calculate the radiomic scores (Rad-scores) and construct a model (Fig. 2F). The model showed an AUC value of 0.77 in the training cohort (Fig. 2G), while the AUC value was 0.68 in the validation cohort. Compared to the ultrasound image-based radiomics model developed by Bove et al. (AUC = 0.756), our radiomics model applied in this study did not achieve a favorable predictive performance. This could be attributed to the high imaging heterogeneity resulting from image acquisition by different operators and machines.

The combined performance of the peripheral blood T-cell model and conventional ultrasound model was evaluated in the validation cohort. The three models were combined in this study to construct a predictive model, and logistic regression analysis demonstrated that all 3 factors were independent predictors of sentinel lymph node metastasis. The combined model achieved an AUC of 0.91 in the training cohort and an AUC of 0.79 in the validation cohort. In the validation cohort, the combined model exhibited a sensitivity of 84.6% and a specificity of 74.1%. Compared to the single-factor model, it demonstrated superior and balanced performance, aligning more closely with the requirements of clinical practice. The clinical decision curve analysis further confirmed that the combined model can yield greater net patient benefit. These values indicated that the combined model could accurately predict sentinel lymph node metastasis in breast cancer patients, which could help in surgical decision-making. Clinical decision curve analysis further demonstrated that compared to univariate predictive models, the combined model resulted in higher net benefits for patients with breast cancer.

This study also presents some limitations. Firstly, the image data that were used for retrospective analysis in this study were acquired by different operators, devices, and probes, resulting in higher levels of random and systematic errors. Secondly, the study needs to be further validated using an external cohort, indicating the need for further improvement in terms of its reliability. Thirdly, the lack of further subtyping data on peripheral blood T cells resulted in a less precise evaluation of the patients’ immune status by the model. In future studies, we plan to further refine the characterization of peripheral blood T cells to obtain a more improved model performance.

In conclusion, ultrasound, as one of the most commonly used clinical imaging modalities, can be used for convenient and non-invasive operations. These specific modeling methods could be employed to predict sentinel lymph node metastasis in breast cancer to some extent. To date, ultrasound radiomics studies for assessing sentinel lymph node metastasis in breast cancer often involve the combination of patient clinical characteristics or immunohistochemical information of the primary tumor. With the inclusion of such diverse factors, the integrated models have shown improved performance. However, our study is the first to propose the use of ultrasound features of the primary breast tumor and peripheral blood T cell subgroups in situations where the pathology is unknown, to construct the integrated model. Furthermore, our study results suggest that, the combined prediction model that comprised the conventional ultrasound, radiomics analysis, and peripheral blood T cell analysis could effectively predict the sentinel lymph node metastasis in breast cancer patients. During the examination of breast cancer patients, ultrasound physicians can significantly enhance their ability to identify sentinel lymph node metastasis in patients with early-stage breast cancer by utilizing the aforementioned combined model. This can provide valuable recommendations for clinical decision-making and improve the patient's overall net benefits. This combined model is non-invasive and carries no surgical risks. And it is a truly surgery-independent model for assessing the presence of metastasis in sentinel lymph nodes in breast cancer. It does not rely on postoperative pathological information but is developed solely based on preoperative ultrasound imaging and peripheral blood samples. It does not require tracer localization, thereby avoiding side effects, such as allergies, trauma, and skin staining. Moreover, it is not affected by operator proficiency, which ensures simple operations with good repeatability. It offers a valuable alternative option for patients who are contraindicated for SLNB or are not suitable for surgery, holding great potential for clinical applications.

## Data Availability

The data used to support the findings of this study are available from the corresponding author upon request.

## Code availability

The authors declare that all data and Python scripts supporting the findings of this study are available from the corresponding author upon reasonable request.

## References

[1] Sung H (2021). Global Cancer Statistics 2020: GLOBOCAN Estimates of Incidence and Mortality Worldwide for 36 Cancers in 185 Countries. CA Cancer J. Clin..

[2] Conti A (2021). Breast cancer. Lancet..

[3] Schrenk P, Wayand W (2001). Sentinel-node biopsy in axillary lymph-node staging for patients with multicentric breast cancer. Lancet..

[4] Kern KA (2002). Concordance and validation study of sentinel lymph node biopsy for breast cancer using subareolar injection of blue dye and technetium 99m sulfur colloid. J. Am. Coll. Surg..

[5] Knauer M (2006). Multicentric breast cancer: A new indication for sentinel lymph node biopsy—a multi-institutional validation study. J. Clin. Oncol..

[6] Tee SR (2018). Meta-analysis of sentinel lymph node biopsy after neoadjuvant chemotherapy in patients with initial biopsy-proven node-positive breast cancer. Br. J. Surg..

[7] de Camargo Teixeira PA, Chala LF, Shimizu C (2017). Axillary lymph node sonographic features and breast tumor characteristics as predictors of malignancy: A nomogram to predict risk. Ultrasound Med. Biol..

[8] Ansari B, Morton MJ, Adamczyk DL (2011). Distance of breast cancer from the skin and nipple impacts axillary nodal metastases. Ann Surg Oncol..

[9] Bae MS, Shin SU, Song SE (2018). Association between US features of primary tumor and axillary lymph node metastasis in patients with clinical T1–T2N0 breast cancer. Acta Radiol..

[10] Yun SJ, Sohn YM, Seo M (2017). Risk stratification for axillary lymph node metastases in breast cancer patients: What clinicopathological and radiological factors of primary breast cancer can predict preoperatively axillary lymph node metastases. Ultrasound Q..

[11] Li XL, Xu HX, Li DD (2017). A risk model based on ultrasound, ultrasound elastography, and histologic parameters for predicting axillary lymph node metastasis in breast invasive ductal carcinoma. Sci. Rep..

[12] Guo Q, Dong Z, Zhang L (2018). Ultrasound features of breast cancer for predicting axillary lymph node metastasis. J. Ultrasound Med..

[13] Zhang H, Sui X, Zhou S (2019). Correlation of conventional ultrasound characteristics of breast tumors with axillary lymph node metastasis and Ki—67 expression in patients with breast cancer. J Ultrasound Med..

[14] Lambin P (2012). Radiomics: Extracting more information from medical images using advanced feature analysis. Eur. J. Cancer.

[15] Li Y (2020). Radiomics with attribute bagging for breast tumor classification using multimodal ultrasound images. J. Ultrasound Med..

[16] Zhang X (2020). Deep learning-based radiomics of B-mode ultrasonography and shear-wave elastography: Improved performance in breast mass classification. Front. Oncol..

[17] Li C (2022). A convolutional neural network based on ultrasound images of primary breast masses: Prediction of lymph-node metastasis in collaboration with classification of benign and malignant tumors. Front. Physiol..

[18] Wu L (2021). Preoperative ultrasound radiomics analysis for expression of multiple molecular biomarkers in mass type of breast ductal carcinoma in situ. BMC Med. Imaging.

[19] Zhou L-Q (2020). Lymph node metastasis prediction from primary breast cancer US images using deep learning. Radiology.

[20] Qiuchang S (2020). Deep learning vs. radiomics for predicting axillary lymph node metastasis of breast cancer using ultrasound images: Don't forget the peritumoral region. Front. Oncol..

[21] Reticker-Flynn NE (2022). Lymph node colonization induces tumor-immune tolerance to promote distant metastasis. Cell.

[22] Harlow SP (2005). Prerandomization Surgical Training for the National Surgical Adjuvant Breast and Bowel Project (NSABP) B-32 trial: A randomized phase III clinical trial to compare sentinel node resection to conventional axillary dissection in clinically node-negative breast cancer. Ann. Surg..

[23] Fanizzi A (2021). Predicting of sentinel lymph node status in breast cancer patients with clinically negative nodes: A validation study. Cancers (Basel).

[24] Bove S (2022). A ultrasound-based radiomic approach to predict the nodal status in clinically negative breast cancer patients. Sci. Rep..

[25] Wu TD (2020). Peripheral T cell expansion predicts tumour infiltration and clinical response. Nature.

[26] Reticker-Flynn NE, Zhang W, Belk JA (2022). Lymph node colonization induces tumor-immune tolerance to promote distant metastasis. Cell.

[27] Engin (2013). Prognostic significance of peripheral blood flow cytometry parameters in patients with non-metastatic breast cancer. Asian Pac. J. Cancer Prev..

